# Internet of Multimedia Things (IoMT): Opportunities, Challenges and Solutions

**DOI:** 10.3390/s20082334

**Published:** 2020-04-20

**Authors:** Yousaf Bin Zikria, Muhammad Khalil Afzal, Sung Won Kim

**Affiliations:** 1 Department of Information and Communication Engineering, Yeungnam University, 280 Daehak-Ro, Gyeongsan, Gyeongbuk 38541, Korea; yousafbinzikria@gmail.com; 2 COMSATS University Islamabad, Wah Campus, Wah Cantt 47010, Pakistan; khalilafzal@ciitwah.edu.pk

**Keywords:** IoT, IoMT, IIoT, WSN, UWSN, ICN, Smart Home, Smart City, VANETS, SDN, Edge Computing

## Abstract

With the immersive growth of the Internet of Things (IoT) and real-time adaptability, quality of life for people is improving. IoT applications are diverse in nature and one crucial aspect of it is multimedia sensors and devices. These IoT multimedia devices form the Internet of Multimedia Things (IoMT). It generates a massive volume of data with different characteristics and requirements than the IoT. The real-time deployment scenarios vary from smart traffic monitoring to smart hospitals. Hence, Timely delivery of IoMT data and decision making is critical as it directly involves the safety of human beings. In this paper, we present a brief overview of IoMT and future research directions. Afterward, we provide an overview of the accepted articles in our special issue on the IoMT: Opportunities, Challenges, and Solutions.

## 1. Introduction

Internet of Things (IoT) devices has limited memory and processing capabilities [1]. Hence, these constrained devices rely upon efficient routing protocols [2] and standardized communication stack [3]. Technological advancements in 5G [4], intelligent 5G-based IoT [5], IoT operating systems (OS) [6], data-driven intelligence in wireless networks [7], scheduling approaches for heterogeneous content-centric IoT [8], congestion avoidance techniques in IoT using data science [9], vehicular ad hoc networks (VANETS) [10], Information-centric networks (ICN) [11], reinforcement learning-based solutions for next-generation networks [12,13], coexistence networks [14], IoT adaptation in agriculture [15] and Healthcare IoT [16] are helping to realize to connect everything and anywhere.

**Figure 1:**
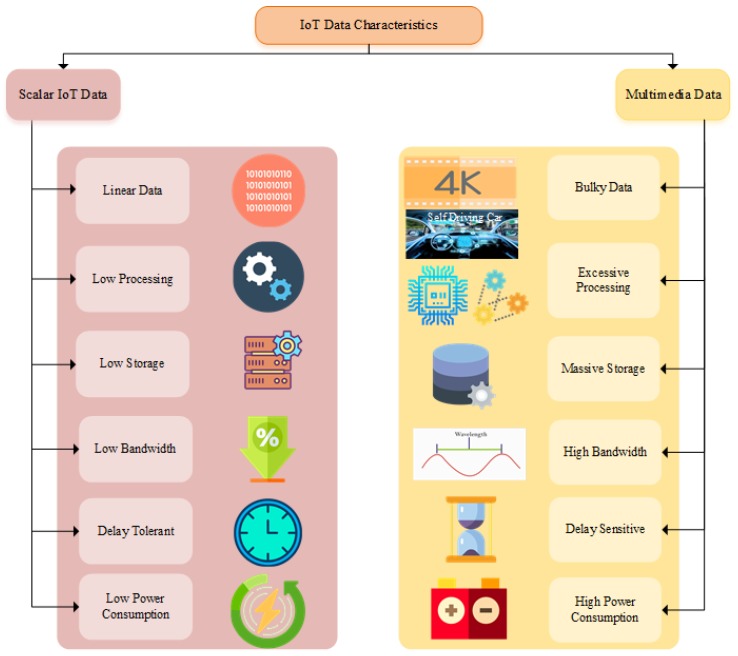
Key Data Characteristics of IoT and IoMT.

Internet of Multimedia Things (IoMT) devices are different from IoT devices. It requires bigger memory, higher computational power, and more power-hungry with higher bandwidth [17]. Figure 1 shows the key data characteristics of IoT and IoMT. The real-time deployment scenarios vary from industrial IoT, Smart cities, Smart hospitals, smart grid, smart agriculture, and smart homes. The main characteristic of IoMT is the timely and reliable delivery of the data. Therefore, it imposes strict quality of service (QoS) requirements and demands efficient network architecture. The users perspective of QoS is known as quality of experience (QoE). QoE can be further characterized as objective or subjective. The users Objective QoE is challenging to measure and dramatically varies according to the needs. However, service providers concern with the subjective QoE to evaluate the network mean opinion score (MOS). The multimedia data is increasing multifold. It raises new challenges to transmit, process, store and share the data. Processing requires new techniques for edge, fog and cloud devices. Further compression and decompression techniques are introduced for the storage of multimedia data. Routing protocol for low-power and lossy networks (RPL) is the standard IoT routing protocol. It needs further development by considering energy-aware, load balancing, fault tolerance, and delay aware IoMT deployment scenarios.

**Figure 2:**
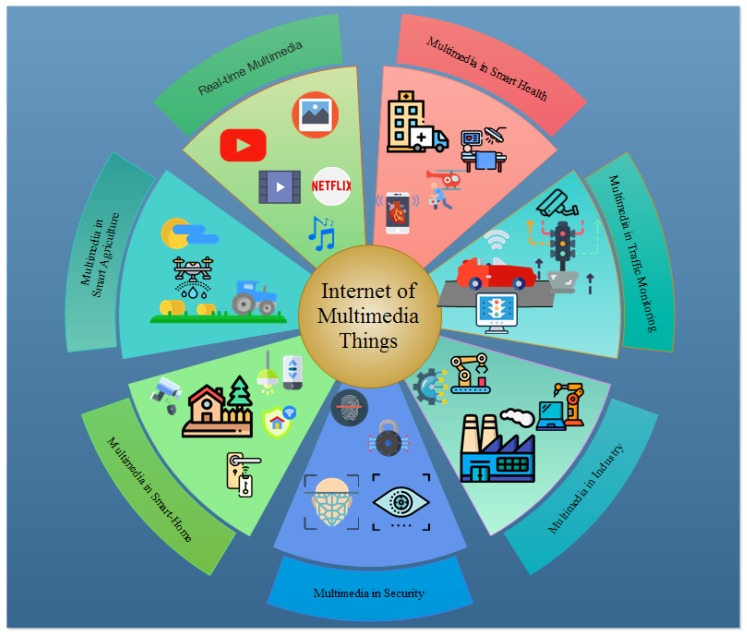
IoMT Applications.

IoT characteristics support multimedia communications; however, multimedia applications are bandwidth-hungry and delay-sensitive. The rapid growth of multimedia traffic in IoT has led the way to innovating new techniques to meet its requirements. IoMT devices require higher bandwidth, bigger memory, and faster computational resources to process data. Typical communications include multipoint-to-point and multipoint-to-multipoint scenarios. Real-world multimedia applications include emergency response systems, traffic monitoring, crime inspection, smart cities, smart homes, smart hospitals, smart agriculture, surveillance systems, Internet of bodies (IoB), and Industrial IoT (IIoT). Dynamic networks, heterogeneous devices and data, strict QoS, and delay sensitivity and reliability requirements over resource-constrained IoMT pose humongous challenges for multimedia communication in IoT. Network-on-chip architecture [18,19] is one of the viable solution to improve the user quality of experience. Figure 2 shows versatile IoMT applications.

The rest of the paper is organized as follows. Section 2 provides future research directions. Section 3 summarizes the accepted paper. Finally, Section 4 concludes the paper.

## 2. Future Research Directions

***Molecular communication*** exploits the transmission and reception of information encoded in molecules. Molecular communications have the potential of becoming the main technology for the execution of advanced medical solutions. The key research challenges in the molecular communication are the interoperability between molecular communication and the other networks, energy-efficient models and protocols for molecular communication, and implantation of reliability in the molecular communication.

***Billions of resource constraint*** devices will be connected in the IoT. The available spectrum is far from enough to support IoT communication systems. Optimal resource allocation for critical multimedia traffic is a key challenge for IoMT. The use of artificial intelligence (machine learning, deep learning) can improve the energy-efficient resource allocation in IoMT.

***Device-to-device (D2D) communication*** in LTE-A will establish direct communication with the device in its communication range. Potential advantages of D2D communication are increased network spectral efficiency, energy efficiency, reduced transmission delay, traffic offloaded base station, and less congestion in the cellular core network. IoMT can take the benefits of the advantages provided by D2D communication. Interference, radio resource allocation, power control, and QoE improvement for cellular users are the key research areas in D2D communication for IoMT.

***Energy Efficient Operation and Protocols*** are the key requirement of IoMT. Many multimedia traffic sources in IoMT may rely on battery-powered sources with limited energy and/or may not be easily accessible for recharging purposes. Similar to WSNs/IoT, the energy-efficient operation, protocols design (i.e., medium access control and routing protocols), and the need to optimize the network lifetime remains a critical challenge for IoMT. Based on the specific application and deployment environment, energy-efficient protocols can be designed for IoMT.

***The Internet of Multimedia Nano-Things*** (IoMNT) is defined as the interconnection of multimedia nano-devices with communication networks and the Internet. The potential applications of IoMNT are security, biomedical, defense, and industry. The main research challenges in IoMNT includes novel medium access control techniques, addressing schemes, neighbor discovery and routing schemes, QoS-aware cross-layer communication module and security solutions for the IoMNT.

***Multimedia-oriented IoT over vehicular networks*** is increasing drastically. Today, vehicles have the capability of supporting real-time acquisition and transmission of the multimedia traffic generated by the built-in IoT devices. However, due to high mobility, density, and random wireless channel conditions, the performance of the delivery of multimedia contents significantly reduces in vehicular networks. Rate adaptation, multimedia delivery over heterogeneous devices, robust video encoding, scalable, and timely delivery of multimedia contents are the key research challenges in IoMT over vehicular networks.

## 3. A Brief Review of Articles of This Special Issue

The immense growth in multimedia traffic over the scarce licensed cellular spectrum has inspired to use unlicensed spectrum below 6 GHz for Long Term Evolution (LTE). However, Wi-Fi uses the same unlicensed band, and this gives rise to the issue of coexistence and fairness of two different technologies in the context of physical and link layer protocols. LTE in the Unlicensed (LTE-U) and LTE License Assisted Access (LTE-LAA) has been proposed in the literature for IoT system. The Third Generation Partnership (3GPP) has standardized LAA for industrial IoT. The coexistence mechanism of LAA follows Listen Before Talk (LBT), which is the same process of Wi-Fi system coexistence i.e., Carrier Sense Multiple Access (CSMA). LTE-U operates a carrier ON/OFF switch policy in duty cycles to maintain fairness in LTE and Wi-Fi transmissions. This mechanism causes spectrum inefficiency. Bajracharya et al. [20] proposed a Machine Learning (ML)-based Adaptive Duty Cycle (ADC) and Dynamic Channel Switch (DCS) mechanism for network to access channel in dynamic network scenarios. ADC and DCS exploit Q-learning to determine the best policy to select an optimal channel and duty cycles. ADC reserve a specific number of sub-frames for Wi-Fi, whereas DCS avoids congested channels for LTE-U users. Performance evaluations are presented in comparison with the fixed duty cycle and channel occupancy time approach. Results show that their proposed method outperforms other methods in the context of fairness and throughput.

With the exponential growth of the IoT, the interaction of multiple physical devices is of extreme importance. These devices are often integrated using Radio Frequency Identification (RFID). The RFID automatically recognizes the object details by reading the physical objects. The system reader, which is equipped with a backend server, uses radio frequencies to communicate with the objects with RFID tags. It makes the practical usage of RFID very vast. Security is the most vital aspect of communication for authentication and securing private data. RFID-based security is beneficial in multiple ways, as RFID does not require a light source and line of sight scenario for communication. Hence RFID can be deployed to sensor monitoring, access control, real-time inventory, and security-aware management systems. However, due to limited computational and memory resources on an RFID tag, limited cryptographic operations can be applied. Therefore, an eavesdropper can forge and access the user’s private data. David et al. in [21] proposed a hash-based RFID authentication mechanism for Context-Aware Sensor Management System (CASMS) to provide security to prevent attacks such as replay, man-in-the-middle, and desynchronization. Hash-based RFID authentication is the five-phase mechanism, namely pre-phase registration, reader pro-tag request and response, tag mutual session key authentication, back end server key authentication, and session key updating. Performance analysis is made based on the Packet Delivery Ratio (PDR) and End-to-End Delay (E2E). Results depict that the proposed model significantly improves PDR and E2E.

Water is the soul of life and essential to the well-being of every person, economy, and the ecosystem on Earth. More than 70% of the Earth is surface is covered by oceans, and they are critical to maintaining the weather and temperature around the globe and providing a means of transportation. However, more than 90% of oceans are unexplored even to the extent that they are still unseen by humans. IoT paved the way to explore and collect the data by connecting different types of networks underwater. Such networks are known as the Internet of Underwater Technology (IoUT) is an emerging technology to support Underwater Sensors Networks (UWSNs) for exploration of undiscovered marine resources. UWSN using communication cables and sensors and maintenance cost is very high. Therefore, underwater wireless communication is proposed. However, UWSN wireless communication is challenging due to environment and propagation losses, which include high noise, Doppler spreading, path loss, multi-path signal propagation, and high power consumption. To overcome these issues, Faheem et al. in [22] proposed cross layered QoS Aware routing Protocol (QoSRP). The proposed scheme is composed of underwater channel detection, channel assignment, and packets forwarding. The QoSRP selects detects the high probability vacant channel and assigns the high data rate channels to an acoustic sensor node. The QoSRP also balances the traffic, avoids congestion, and data path loops to increase PDR and throughput of the system with minimum delay along the path.

Ultra Wide band (UWB) features include higher bandwidth, and it is one of the viable technologies for IoMT applications. The integration of UWB in health critical IoT applications can provide an effective and reliable solution for the monitoring of patients. Ataxia patients suffer from abnormal movement, and that severely affects walking activities. The walking activities can be classified as a normal walk, difficulty walking in a straight line, walking with heavy steps, and forward bending walking. All of these walking patterns except normal walking shows abnormality. Zilani et al. [23] proposed a scheme to cater to this problem. They set up the testbed in an indoor environment. They collected sample walk patterns and classified it using Support Vector Machine (SVM) algorithms, namely SVM-based Sigmoid Kernel Function (SKF) and Radial Basis Function (RBF). Results show that RBF performs better than SKF. The drawback of the proposed scheme is that it is only tested for a single person. Hence, it should be extended to monitor multiple persons.

Security, privacy, and trust remain challenges in IoMT because of the openness and heterogeneity of IoMT. Access control is used to protect the confidentiality and integrity of constrained resources in IoMT services. It provides a solution to avoid any unauthorized access for multimedia applications in IoT services. However, due to increasing the number of users and multimedia services offered by the IoT platform, the access control system is becoming more and more multifaceted. Besides, access control policy evaluation reduces the performance of IoMT applications. Therefore, Meiping Liu et al. [24] proposed an Attribute-Based Access Control (ABAC) policy retrieval method to improve the performance of access control policy evaluation in multimedia networks. To rebuild the policy decision tree, an attribute value level, and the depth index is introduced, thus, improve policy retrieval efficiency. Policy analysis is performed with a different number of rules and the increasing complexity of the policy. Results indicate that the proposed method is more efficient and scalable than the existing access control schemes.

Increasing demand for data-intensive applications is growing users’ data requirements exponentially. However, spectrum scarcity is the biggest hurdle in meeting users QoS. One of the viable solutions is to reuse and share the spectrum among the users to fulfill users demands without compromising the user’s experience. Even though unlicensed spectrums are available for free, they are already overcrowded. Different network technologies such as 5G and Wi-Fi use different spectrum access mechanisms, and sharing the spectrum among them is a trivial task. To allow fair coexistence between 5G and Wi-Fi networks operating in the same spectrum, LBT is introduced to work in parallel with the CSMA for channel access. RL techniques can be adapted to make a spectrum access mechanism to learn network conditions itself and adapt to the network changes accordingly. Consequently, the network becomes sustainable and self-adaptive. Neto et al. in [25] proposed Q-Learning to LTE-U to adjust the duty cycle parameters to reduce coexistence interference and improve the system data rate. A saturated network scenario is considered to evaluate the proposed scheme in the ns-3 simulator. The proposed algorithm performs well in a multi-cell coexistence network scenario. Hence, it improves overall system performance by achieving a higher data rate for users and systems compared to the existing conventional mechanism.

## 4. Conclusions

Six papers in this SI presented state-of-the-art research trend in the area of IoMT opportunities, challenges, and solutions. The papers presented an interesting discussion and novel ideas for the readers. The guest editors would like to show appreciation to the authors and thank all the anonymous reviewers for providing constructive feedback to improve the overall quality of all the accepted papers. We would also like to thank sensors Editor in Chief Prof. Dr. Vittorio M.N. Passaro, Associate Editor in Chief of the IoT section Prof. Dr. Raffaele Bruno, and managing editor Missy Wu for the invaluable help and productive advice in finalizing this SI.

## Abbreviations

The following abbreviations are used in this manuscript:

ADC	Adaptive Duty Cycle
ABAC	Attribute-Based Access Control
CSMA	Carrier Sense Multiple Access
CASMS	Context-Aware Sensor Management System
DCS	Dynamic Channel Switch
E2E	End-to-End Delay
IoT	Internet of Things
IIoT	Industrial IoT
ICN	Information-centric networks
IoB	Internet of bodies
IoMNT	Internet of Multimedia Nano-Things
(IoMT)	Internet of Multimedia Things
IoUT	Internet of Underwater Technology
LBT	Listen Before Talk
LTE	Long Term Evolution
LTE-U	LTE in the Unlicensed
LTE-LAA	LTE License Assisted Access
ML	Machine Learning
MoS	Mean Opinion Score
OS	Operating Systems
PDR	Packet Delivery Ratio
QoSRP	QoS Aware routing Protocol
QoE	Quality of Experience
QoS	Quality of Service
RBF	Radial Basis Function
RFID	Radio Frequency Identification
RPL	Routing protocol for low-power and lossy networks
SKF	Sigmoid Kernel Function
SVM	Support Vector Machine
3GPP	Third Generation Partnership
UWB	Ultra Wide band
VANETS	Vehicular Ad Hoc Networks

## References

[1] Zikria Y.B., Kim S.W., Hahm O., Afzal M.K., Aalsalem M.Y. (2019). Internet of Things (IoT) Operating Systems Management: Opportunities, Challenges, and Solution. Sensors.

[2] Zikria Y.B., Afzal M.K., Ishmanov F., Kim S.W., Yu H. (2018). A survey on routing protocols supported by the Contiki Internet of things operating system. Future Gener. Comput. Syst..

[3] Zikria Y.B., Yu H., Afzal M.K., Rehmani M.H., Hahm O. (2018). Internet of Things (IoT): Operating System, Applications and Protocols Design, and Validation Techniques. Future Gener. Comput. Syst..

[4] Zikria Y.B., Kim S.W., Afzal M.K., Wang H., Hahm O., Rehmani M.H. (2018). 5G Mobile Services and Scenarios: Challenges and Solutions. Sustainability.

[5] Afzal M.K., Zikria Y.B., Mumtaz S., Rayes A., Al-Dulaimi A., Guizani M. (2018). Unlocking 5G Spectrum Potential for Intelligent IoT: Opportunities, Challenges, and Solutions. IEEE Commun. Mag..

[6] Musaddiq A., Zikria Y.B., Hahm O., Yu H., Bashir A.K., Kim S.W. (2018). A Survey on Resource Management in IoT Operating Systems. IEEE Access.

[7] Afzal M.K., Zikria Y.B., Ni Q. (2019). Data-driven intelligence in wireless networks: Issues, challenges, and solution. Trans. Emerg. Tel. Tech..

[8] Al-Turjman F., Ever E., Zikria Y.B., Kim S.W., Elmahgoubi A. (2019). SAHCI: Scheduling Approach for Heterogeneous Content-Centric IoT Applications. IEEE Access.

[9] Kazmi H.S.Z., Javaid N., Awais M., Tahir M., Shim S., Zikria Y.B. (2019). Congestion avoidance and fault detection in WSNs using data science techniques. Trans. Emerg. Telecommun. Technol..

[10] Rasool I.U., Zikria Y.B., Kim S.W. (2017). A review of wireless access vehicular environment multichannel operational medium access control protocols: Quality-of-service analysis and other related issues. Int. J. Distrib. Sens. Netw..

[11] Meng Y., Naeem M.A., Ali R., Zikria Y.B., Kim S.W. (2019). DCS: Distributed Caching Strategy at the Edge of Vehicular Sensor Networks in Information-Centric Networking. Sensors.

[12] Ali R., Shahin N., Zikria Y.B., Kim B., Kim S.W. (2018). Deep Reinforcement Learning Paradigm for Performance Optimization of Channel Observation–Based MAC Protocols in Dense WLANs. IEEE Access.

[13] Ali R., Ali N., Zikria Y.B., Kim B., Kim S.W. (2019). Performance optimization of QoS-supported dense WLANs using machine-learning-enabled enhanced distributed channel access (MEDCA) mechanism. Neural Comput. Appl..

[14] Bajracharya R., Shrestha R., Zikria Y.B., Kim S.W. (2018). LTE in the Unlicensed Spectrum: A Survey. IETE Tech. Rev..

[15] Nauman A., Qadri Y.A., Amjad M., Zikria Y.B., Afzal M.K., Kim S.W. (2020). Multimedia Internet of Things: A Comprehensive Survey. IEEE Access.

[16] Farooq M.S., Riaz S., Abid A., Umer T., Zikria Y.B. (2020). Role of IoT Technology in Agriculture: A Systematic Literature Review. Electronics.

[17] Qadri Y.A., Nauman A., Zikria Y.B., Vasilakos A.V., Kim S.W. (2020). The Future of Healthcare Internet of Things: A Survey of Emerging Technologies. IEEE Commun. Surv. Tutor..

[18] Ibrahim M., Baloch N.K., Anjum S., Zikria Y.B., Kim S.W. An Energy Efficient and Low Overhead Fault Mitigation Technique for Internet of Thing Edge Devices Reliable on-Chip Communication. Software: Practice and Experience. https://onlinelibrary.wiley.com/doi/pdf/10.1002/spe.2796.

[19] Shafique M.A., Baloch N.K., Baig M.I., Hussain F., Zikria Y.B., Kim S.W. (2020). NoCGuard: A Reliable Network-on-Chip Router Architecture. Electronics.

[20] Bajracharya R., Shrestha R., Kim S.W. (2019). Q-Learning Based Fair and Efficient Coexistence of LTE in Unlicensed Band. Sensors.

[21] Deebak B.D., Al-Turjman F., Mostarda L. (2019). A Hash-Based RFID Authentication Mechanism for Context-Aware Management in IoT-Based Multimedia Systems. Sensors.

[22] Faheem M., Butt A.R., Raza B., Alquhayz H., Ashraf W.M., Shah B.S., Ngadi A.M., Gungor C.V. (2019). QoSRP: A Cross-Layer QoS Channel-Aware Routing Protocol for the Internet of Underwater Acoustic Sensor Networks. Sensors.

[23] Zilani T.A., Al-Turjman F., Khan M.B., Zhao N., Yang X. (2020). Monitoring Movements of Ataxia Patient by Using UWB Technology. Sensors.

[24] Liu M., Yang C., Li H., Zhang Y. (2020). An Efficient Attribute-Based Access Control (ABAC) Policy Retrieval Method Based on Attribute and Value Levels in Multimedia Networks. Sensors.

[25] Neto D.M.J., Neto G.F.S., Santana M.P., Junior D.A.V. (2020). Multi-cell LTE-U/Wi-Fi coexistence evaluation using a reinforcement learning framework. Sensors.

